# Ehlers–Danlos syndrome type IV: a case report of a rare cause of spontaneous sigmoid perforation and enteroatmospheric fistulae in a child

**DOI:** 10.1186/s40792-020-01051-0

**Published:** 2020-12-08

**Authors:** Hemal Kodikara, Sebastian K. King, Elizabeth McLeod

**Affiliations:** 1 Department of Paediatric Surgery, The Royal Children’s Hospital, Melbourne, Australia; 2 Department of Paediatric Surgery, Christchurch Hospital, Christchurch, New Zealand; 3 Department of Paediatrics, University of Melbourne, Melbourne, Australia; 4 Surgical Research, Murdoch Children’s Research Institute, Melbourne, Australia

**Keywords:** Ehlers–Danlos syndrome type IV, Sigmoid perforation, Negative pressure wound therapy, Enteroatmospheric fistula, Paediatric

## Abstract

**Background:**

Ehlers–Danlos syndrome (EDS) type IV is a rare subtype of EDS, but has important surgical implications.

Case presentation

Here, we present a case of a spontaneous sigmoid perforation in a 14-year-old boy. He was initially treated with laparotomy, oversew of the sigmoid perforation and a diverting ileostomy. He developed a complete wound dehiscence and enteroatmospheric fistulae. These were managed with a combination of negative pressure wound therapy and Eakin (TG Eakin™) pouch changes. We discuss the clinical features and presentation of EDS type IV, the surgical implications of managing patients with the condition, and the challenges in management of enteroatmospheric fistulae in children.

**Conclusions:**

Ehlers–Danlos syndrome type IV should be considered as a cause of any spontaneous colonic perforation in children.

## Background

Ehlers–Danlos syndrome (EDS) is a heterogeneous group of hereditary connective tissue diseases, characterised by abnormalities in the joints, skin and connective tissue. Type IV is a rare subtype of EDS, uncommonly encountered in clinical practice, with an incidence of approximately 1/200,000. Joint hypermobility is not usually a major feature; instead, it is characterised by distinctive facial features, translucent skin with visible subcutaneous vessels on the trunk, easy bleeding/bruising and severe arterial, gastrointestinal and uterine complications, not seen in the other forms of EDS. We discuss the presentation and management of an adolescent patient who presented with spontaneous colonic perforation, who went on to develop multiple and refractory enteroatmospheric fistulae.

## Case presentation

A 14-year-old boy, with no significant past medical or family history, was assessed at our tertiary paediatric surgical centre following a 3-day history of abdominal pain, vomiting and fever. Examination revealed a patient in septic shock with generalised abdominal rigidity. A chest radiograph demonstrated a moderate volume of sub-diaphragmatic air. Following resuscitation and initiation of intravenous antibiotics, diagnostic laparoscopy demonstrated widespread faecal peritonitis and a midline laparotomy was performed. A 3-mm perforation of the proximal sigmoid colon was demonstrated, with no evidence of diverticulum nor other colonic abnormality. The procedure was complicated by extensive bleeding from the tissues and an iatrogenic defect in the ileal mesentery, resulting in ischaemia of 10 cm of terminal ileum. Given this, the decision was made to oversew the sigmoid perforation with interrupted PDS sutures, resect and anastomose the ischaemic ileum, and perform a defunctioning loop ileostomy. The patient was transferred to the intensive care unit, where he made a satisfactory recovery and was extubated on day 3.

**Fig. 1:**
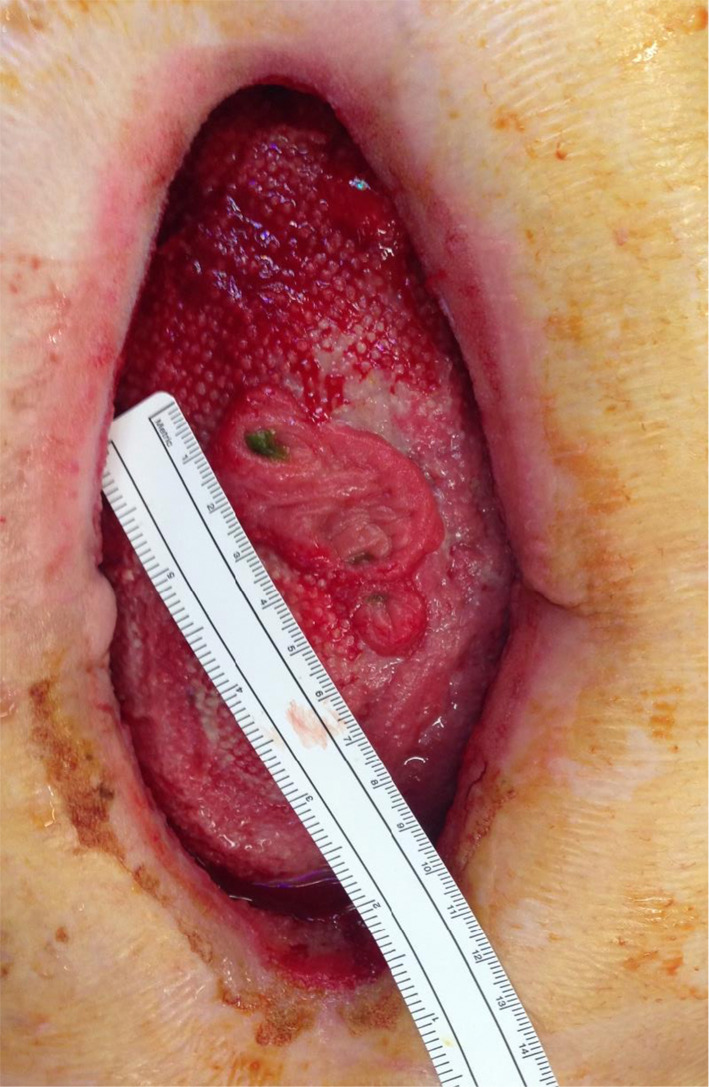
Early appearance of abdominal wall with fistulae demonstrated

From the third postoperative day, serous fluid was noted on the abdominal dressing; however, the wound remained intact. He was transferred to the ward, ileostomy function commenced and enteral feeding started. On postoperative day 9, he was noted to have a complete wound dehiscence and was taken back to theatre for assessment. There was wide separation of the fascial edges, with exposed intestine. The wound was cleaned and a negative pressure dressing applied, with Mepitel (Molnlycke™) used as the interface between the sponge and the intestine. On postoperative day 11, small bowel effluent was noted in the suction container. In theatre, four small bowel fistulae in a superficial bowel loop were demonstrated (Fig. 1), and a negative pressure dressing was reapplied to manage the new fistulae. A literature search suggested a phenotype of EDS type IV, including the characteristic facial features, acrogeria, translucent skin, and other less common features, such as nocturnal lagophthalmos (sleeping with eyelids open). Genetic testing demonstrated a COL3A1 mutation, confirming the diagnosis of EDS type IV.

**Fig. 2:**
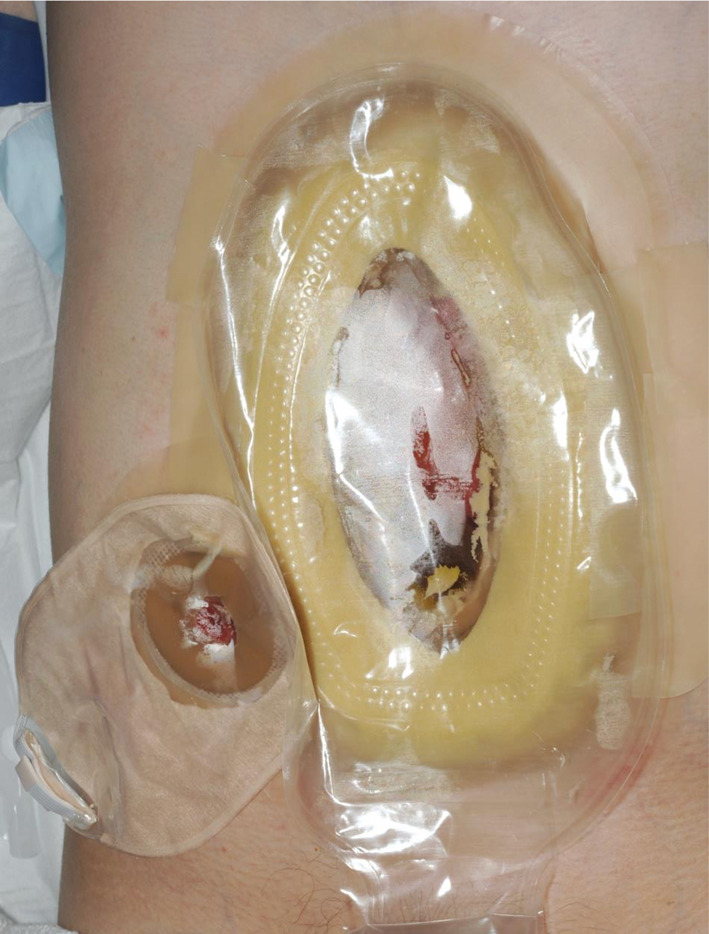
Use of large custom-fit stoma pouch to midline fistulae

**Fig. 3:**
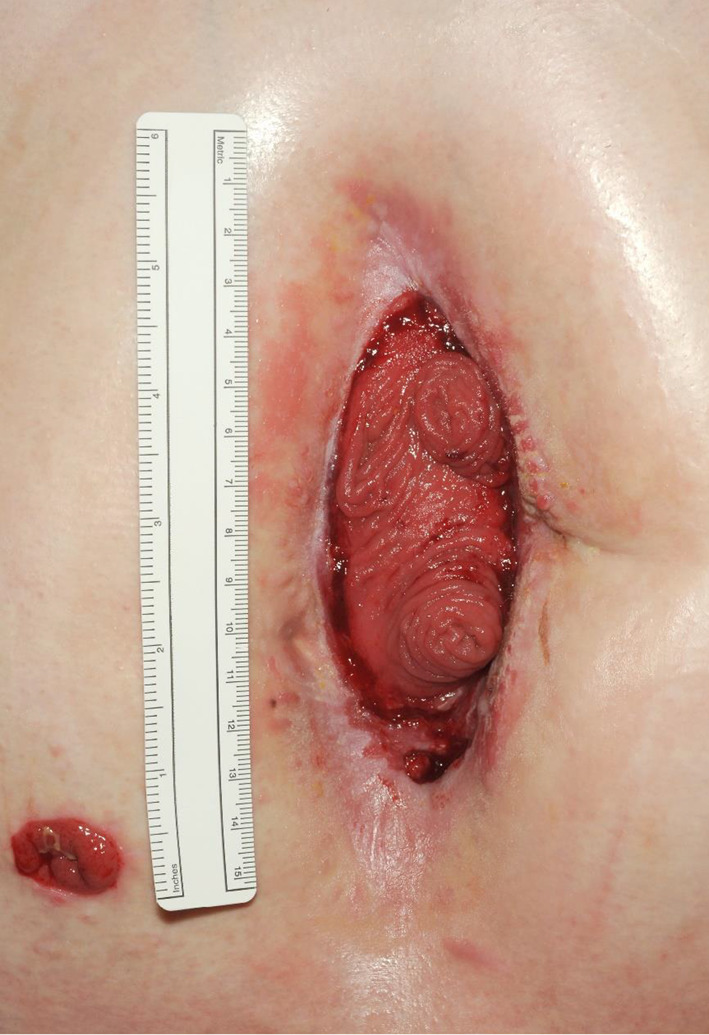
Later appearance demonstrating the complex midline jejunostomy (note ileostomy in right iliac fossa)

Negative pressure dressing changes occurred every 48–72 h to allow the wound to granulate. The following 2 months involved mobilisation, introduction of oral intake, and maturation and eversion of the fistulae (over a period of 4 weeks). Imaging, including CT with oral contrast, contrast follow-through studies and stomagram, were used to define the fistulae in the distal jejunum. Examination under anaesthesia confirmed that all four fistulae within the same bowel loop. Despite considerable efforts, the negative pressure dressings were ineffectual at removing the effluent from the wound, and it became clear that closure of the fistulae and abdominal wall would not be possible. This led to a change in management, with the use of a large custom-fit pouch stoma bag (TG Eakin Ltd), encompassing the entire midline wound and fistulae (Fig. 2). Dressing changes were performed every 48–96 h under general anaesthesia due to pain levels. The fistula output was highly variable (between 400 and 3000 ml daily depending on oral intake). High output from the fistula did become a significant problem requiring intravenous replacement and additional TPN supplementation, but this was ameliorated with loperamide, pectin, Lomotil (atropine and diphenoxylate) and adjusted feed rates and volumes. Control of sepsis was never an issue for this patient, however he required multiple blood transfusions, with presumed occult GI blood loss. Over the following 4 months the wound contracted significantly in size, and he was able to tolerate nasogastric feeding without significant increases in stomal outputs. The patient was discharged 6 months postoperatively, with nocturnal parenteral nutrition and thrice weekly stoma bag changes, with the enteroatmospheric fistula having matured into a complex midline jejunostomy (Fig. 3).

Management of the patient’s stoma continue to be problematic (skin breakdown, pain, psychological distress), resulting in the decision to attempt repair of the fistulae. Following nutritional optimisation, a further laparotomy was performed. This was technically challenging due to dense adhesions, multiple serosal tears, significant oozing, and abnormalities in the tensile strength of the small bowel. Five jejunal fistulae within a short segment (~ 15 cm) were mobilised and resected, with one resulting anastomosis and good preservation of small bowel length. A sheet of Vicryl mesh was placed into the defect, and 2 Jackson Pratt drains overlain. A negative pressure dressing was overlain and the laparostomy progressively closed over a period of 1 month with drains left in situ. Unfortunately, there was further enteric leakage 1 month post-resection, and the patient was discharged 4 months after the laparotomy, with close surveillance by clinical nutrition, endocrinology and adolescent psychiatry teams.

Over the following 6 months, a transition to stoma bag changes under sedation was achieved. The patient developed osteopenia, with multiple compression fractures of the thoracolumbar spine, and a shaft of humerus fracture which complicated long-term pain management goals. Dependence on analgesia, oppositional defiance disorder and depression developed. After discussion with the family, a decision was made to manage the fistulae conservatively. Eighteen months after re-fistulation, the family are managing the stoma well at home. Mood and social functioning have also improved.

Spontaneous sigmoid perforation is rare in children, and potential causes include foreign body, stercoral, diverticular disease, and inflammatory bowel disease. Spontaneous sigmoid perforation has been described most often in young children and infants in the setting of febrile illness (especially gastroenteritis or respiratory tract infection), current hospitalisation and abdominal distension [1]. Our adolescent patient had no known underlying illness nor risk factors for any of these conditions, and so consideration was given to rarer potential causes, such as EDS type IV.

The Ehlers–Danlos syndromes are a group of hereditary abnormalities of connective tissue, whose prevalence is estimated between 1/10,000 and 1/25,000, with no ethnic predilection [2]. EDS type IV comprises 5–10% of all EDS patients and is an autosomal dominant disorder caused by a mutation in the type III collagen gene, COL3A1. Arterial walls and the walls of the digestive tract are rich in type III collagen, which leads to fragility of these structures, predisposing to spontaneous rupture [3]. As 25% of patients with EDS type IV experience their first major complication by the age of 20 years, it is of vital importance to the paediatric surgeon to be aware of the condition [4] (Table 1).

**Table 1:** Clinical diagnosis of vascular Ehlers–Danlos syndrome is based on the Villefranche criteria [5]

Major criteria	Minor criteria
Arterial, digestive or uterine fragility or rupture	Positive family history or sudden death in a close relative
Thin, translucent skin	Acrogeria
Extensive bruising	Hypermobility of small joints
Characteristic facial appearance	Tendon and muscle rupture
	Talipes equinovarus
	Early-onset varicose veins
	Spontaneous pneumothorax or haemothorax

**Fig. 4:**
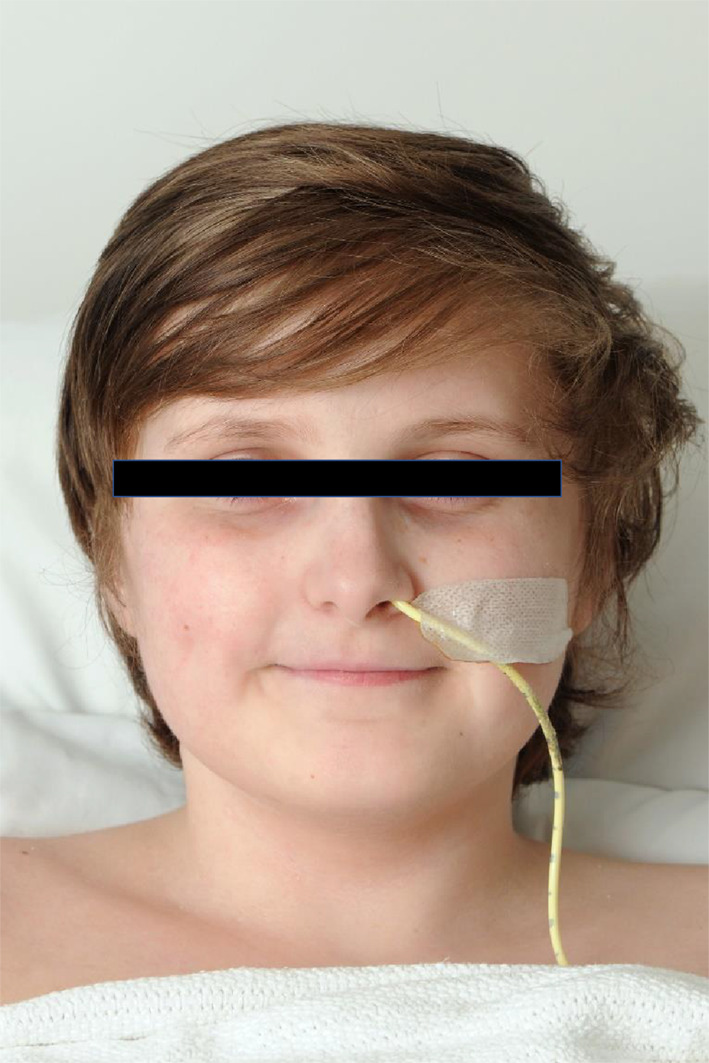
Characteristic facial features of EDS type IV (prominent eyes, thin pointed nose, narrow lips)

Our patient had 3/4 major criteria, including the characteristic facial features (Fig. 4) and 2/7 minor criteria. A definitive diagnosis may be achieved by obtaining cultured dermal fibroblasts showing abnormalities of type III procollagen production, or the more recently developed technique of molecular genetic testing of COL3A1 [2].

Spontaneous sigmoid perforation in the setting of EDS type IV has been previously reported in the literature [6, 7], from as early as age 5 through to adulthood [3].The high levels of collagen found in the colon give this particular site a greater propensity for involvement compared with the small intestine [8]. However, there is no consensus over either the initial surgical management or the long-term management of the colon, which is at high risk of re-perforation [8]. One retrospective review evaluated 41 colonic perforations, 80% of which were in the sigmoid colon. Of these patients, 66% were treated with resection and diversion, with 18 eventually undergoing restoration of intestinal continuity. A total of 55.6% of these patients suffered a re-perforation, some with three or four perforations [9].

Our patient had a complicated postoperative course, with multiple factors potentially contributing to his wound dehiscence and subsequent enteric fistulation. These included severe peritonitis, profound systemic compromise, and his underlying connective tissue disorder. Patient with EDS type IV are reported to have high rates of surgical and postoperative complications [9]. General surgical complications include excessive bleeding (due to capillary fragility rather than clotting dysfunction), poor tissue quality, and impaired wound healing (tissue tears with minimal handling, fistula formation, fascial dehiscence, and incisional herniation) [10].

The use of negative pressure wound therapy (NPWT) for paediatric laparostomy has been published widely and is used for a range of conditions [11, 12]. NPWT has been demonstrated to have major benefits: protection against external damage and bacterial ingress; management of fluids, analgesia, and skin protection; and closure of the abdomen [13]. However, a longstanding concern with NPWT is whether it promotes enteric fistulation. This has been largely discounted by recent evidence from a large nationwide audit in an adult population in the United Kingdom which demonstrated that the risk was no higher than when using conventional wound therapies [14]. Nevertheless, enteric fistulae may form, and this risk appears to be higher in patients with an underlying septic condition [13]. Enteroatmospheric fistulae are notoriously difficult to manage, with high rates of associated generalised sepsis, central venous difficulties, and mortality approaching 40% [15]. Since spontaneous closure is rare and attempts at early primary closure are almost always unsuccessful, numerous methods have been devised to manage the fistula effluent until such time-definitive surgical closure may be attempted. The aims of early enteric fistula management include prevention of sepsis, diversion of fistula output, and correction of any fluid and electrolyte imbalances. These methods include NPWT (including various techniques to isolate the fistula), biologic dressings/fibrin and cyanoacrylate glues, patches/plugs/flaps, and floating stomas. All have been shown to have varying success rates [15].

We utilised a Mepitel (Molnlycke™) interface between the NPWT sponge and the intestine and used a very low pressure setting (40 mmHg) in order to minimise any possible trauma to the intestine. However, if the diagnosis of EDS type IV had been known during the initial laparotomy, an alternative wound therapy system may have been more appropriate. The NPWT worked effectively to manage the fistulae in the early weeks, with excellent control of the effluent, no episodes of sepsis and good condition of the skin. This method of wound management has been described extensively in the adult but not paediatric literature, and has been shown to result in a spontaneous fistula closure rate of 61% [16]. However, as oral intake was established and the NPWT occluded and became less effective at removing the effluent, a large custom-fit pouch stoma bag (TG Eakin Ltd™) proved to be an effective way of managing the large wound and fistulae.

## Conclusions

Whilst rare, Ehlers–Danlos syndrome type IV must be considered in children who present with spontaneous visceral (especially sigmoid) perforation. Awareness of this subtype of EDS is important, as patients with EDS type IV often do not feature the classic hypermobility and joint laxity associated with the more commonly known, classical subtypes. Furthermore, spontaneous visceral perforation may be the first presentation of the condition. As the mutation is spontaneous in 50% of patients there may not be a family history of the disorder. Therefore, the clinical findings of the condition, such as characteristic facial features, translucent skin and easy bruising, should be sought in patients with spontaneous colonic perforation without a clear cause.

Enteroatmospheric fistulae are not commonly reported in children and are very difficult to manage. We developed good control of the effluent, remained free of sepsis complications and obtained good care of the skin with a combination of negative pressure dressing and use of large custom-fit pouch stoma bags. Unfortunately, the long-term management of the colon remains unclear in patients with EDS type IV, given the high rate of recurrent perforations in patients.

## Data Availability

Copies of the manuscript, figures and tables are held with author HK.

## Abbreviation

EDS: Ehlers–Danlos syndrome
